# Effect of dapagliflozin on diabetic patients with cardiovascular disease via MAPK signalling pathway

**DOI:** 10.1111/jcmm.16786

**Published:** 2021-07-14

**Authors:** Zhaodi Yue, Li Li, Hui Fu, Yanyan Yin, Bingyu Du, Fangqi Wang, Yi Ding, Yibo Liu, Renjie Zhao, Zhongwen Zhang, Shaohong Yu

**Affiliations:** ^1^ Department of rehabilitation medicine Department of Endocrinology and Metabology The Second Affiliated Hospital of Shandong University of Traditional Chinese Medicine The First Affiliated Hospital of Shandong First Medical University & Shandong Provincial Qianfoshan Hospital Jinan China; ^2^ The Second Clinical Medical College Shandong University of Traditional Chinese Medicine Jinan China; ^3^ Department of Endocrinology and Metabology The First Affiliated Hospital of Shandong First Medical University & Shandong Provincial Qianfoshan Hospital Shandong Key Laboratory of Rheumatic Disease and Translational medicine Shandong Institute of Nephrology Jinan China; ^4^ College of Rehabilitation Medicine Shandong University of Traditional Chinese Medicine Jinan China; ^5^ The Clinical Medical College Cheeloo Medical College of Shandong University Jinan China

**Keywords:** cardiovascular disease, dapagliflozin, network pharmacology, type 2 diabetes mellitus

## Abstract

Clinical studies have shown that dapagliflozin can reduce cardiovascular outcome in patients with type 2 diabetes mellitus (T2DM), but the exact mechanism is unclear. In this study, we used the molecular docking and network pharmacology methods to explore the potential mechanism of dapagliflozin on T2DM complicated with cardiovascular diseases (CVD). Dapagliflozin's potential targets were predicted via the Swiss Target Prediction platform. The pathogenic targets of T2DM and CVD were screened by the Online Mendelian Inheritance in Man (OMIM) and Gene Cards databases. The common targets of dapagliflozin, T2DM and CVD were used to establish a protein‐protein interaction (PPI) network; the potential protein functional modules in the PPI network were found out by MCODE. Metascape tool was used for Gene Ontology (GO) and Kyoto Encyclopaedia of Genes and Genomes (KEGG) pathway enrichment analysis. A potential protein functional module with the best score was obtained from the PPI network and 9 targets in the protein functional module all showed good binding properties when docking with dapagliflozin. The results of KEGG pathway enrichment analysis showed that the underlying mechanism mainly involved AGE‐RAGE signalling pathway in diabetic complications, TNF signalling pathway and MAPK signalling pathway. Significantly, the MAPK signalling pathway was considered as the key pathway. In conclusion, we speculated that dapagliflozin played a therapeutic role in T2DM complicated with CVD mainly through MAPK signalling pathway. This study preliminarily reveals the possible mechanism of dapagliflozin in the treatment of T2DM complicated with CVD and provides a theoretical basis for future clinical research.

## 1 INTRODUCTION

Type 2 diabetes mellitus (T2DM) is a metabolic disease caused by multiple aetiologies. The prevalence of T2DM is rising year by year, which has become the primary disease threatening the health and life of human beings.^1^, ^2^ Hyperglycaemia is a well‐established risk factor for cardiovascular diseases (CVD).^3^ At present, T2DM patients complicated with CVD were treated with hypoglycaemic, hypotensive and lipid‐lowering therapy, but these therapies have not effectively reduced adverse cardiovascular events and the curative effect is not satisfactory. Therefore, there is an urgent need to explore new therapeutic drugs.

Dapagliflozin, a SGLT2 inhibitor, is a novel hypoglycaemic drug and has been widely used in the treatment of T2DM. A number of large double‐blind clinical studies have demonstrated that dapagliflozin reduced the composite end‐point of cardiovascular hospitalization and death in patients with T2DM.^3^, ^4^ However, the exact mechanism of dapagliflozin on improvement cardiovascular outcome in patient with T2DM is still unclear.

Drug targeting interaction is the basis of target discovery of therapeutic drugs.^5^ Over the past decade, basic researches have been used to explore the potential therapeutic targets of drugs for diseases, but these methods are time‐consuming and expensive. With the rapid development of systems biology and computer network, various computational methods have been proposed to predict potential therapeutic targets of drugs for diseases, such as molecular docking‐based, network‐based and pharmacophore‐based methods^6^, ^7^, ^8^; based on the advantages of high efficiency and low cost, more and more researches have adopted computational methods to predict the drug targeting interaction. Therefore, in this study, we performed the network pharmacology to explore the potential therapeutic targets and signalling pathways of dapagliflozin on T2DM complicated with CVD, using molecular docking method to predict the recognition and interaction modes between dapagliflozin and its candidate targets.

## 2 MATERIALS AND METHODS

### 2.1 Screening of dapagliflozin's potential targets

The bioactive component of dapagliflozin was searched from the published literature in PubMed database, with “dapagliflozin” as the key word. Then, the structural formula of bioactive component retrieved in the literature was checked with that in the chemical database (http://www.chemsrc.com/). Based on the method that similar drugs share similar targets,^5^ potential targets of dapagliflozin were predicted with Swiss Target Prediction (http:// www.swisstargetprediction.ch/). ^9^ The species was limited to ‘Homo sapiens’, and the targets of probability >0.1 were finally selected as the prediction results. To standardize the target information, the target names were standardized in the Uniprot protein database (http://www.uniprot.org/). ^10^

### 2.2 Screening of targets for T2DM complicated with CVD

The pathogenic targets of T2DM complicated with CVD were searched by Online Mendelian Inheritance in Man (OMIM, http://omim.org/) ^11^ and Gene Cards (http://www.genecards.org/) databases,^12^ using the terms “type 2 diabetes mellitus” in combination with “coronary heart disease” or “cardiovascular diseases” or “heart failure” or “myocardial remodelling” or “atherosclerosis”. Because the higher score of interaction between genes and diseases means that the target is more closely related to the disease. Therefore, we selected genes with score higher than median as candidate targets from the Gene Cards database.

### 2.3 Construction of protein‐protein interaction (PPI) network

In order to identify the interaction targets of dapagliflozin in the treatment of T2DM complicated with CVD, we selected the online drawing tool Interactive Venn (http://www.interactivenn.net/) ^13^ to draw a Venn diagram, whose overlapping section represented the common targets for dapagliflozin and T2DM complicated with CVD. These common targets were submitted to STRING11.0 platform (http://string‐db.org/), ^14^ and the PPI network was obtained after the disconnected nodes were hidden. Then MCODE,^15^ a cluster analysis plug‐in of Cytoscape3.7.2 (http://cytoscape.org/), ^16^ was utilized to further analyse the PPI network. The targets in the protein functional module with the best score analysed by the MCODE were selected as core targets for further molecular docking.

### 2.4 Enrichment analysis

Gene Ontology (GO) functional analysis and Kyoto Encyclopaedia of Genes and Genomes (KEGG) pathway enrichment analysis were performed using Metascape (https://metascape.org/),^17^ with *P* <.01 as the cut‐off criteria. The top 10 GO items and top 20 KEGG pathways that meet the criterion were visualized by bioinformatics online tools (http://www.bioinformatics.com.cn/) and EHBIO Gene Technology Platform (http://www.ehbio.com/ImageGP/).

### 2.5 Construction of component‐target‐pathway network

Cytoscape3.7.2 was used to construct a component‐target‐pathway network based on the data of KEGG enrichment analysis, and the topology parameters of the network were analysed with the built‐in tool Network Analyzer in Cytoscape.

### 2.6 Molecular docking verification

**FIGURE 1:**
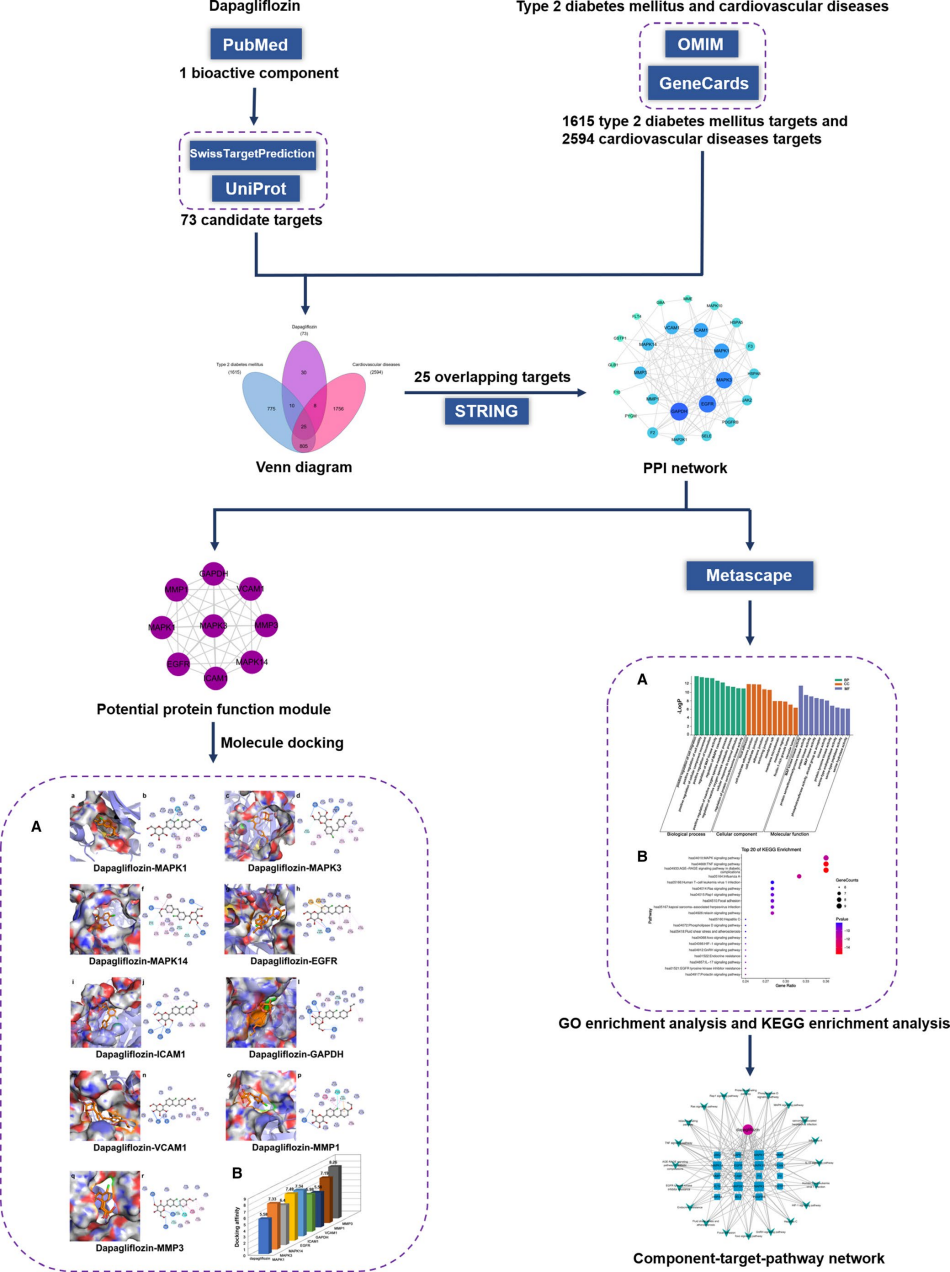
Flow chart of investigating dapagliflozin in the treatment of T2DM complicated with CVD

The “sdf” file of dapagliflozin bioactive component was downloaded from the PubChem database (https://pubchem.ncbi.nlm.nih.gov/), ^18^ and its mechanical structure was optimized by Open Babel2.4.0.^19^ Appropriate 3D structure “pdb” files of the core targets were then downloaded from RCSB Protein Data Bank (http://www.rcsb.org/). ^20^ AutoDockTools1.5.6 ^21^ was used to perform the molecular docking to verify the binding affinity between targets and bioactive component of dapagliflozin. The binding conformations were visualized by PyMOL2.4.0^22^ and Discovery Studio4.5. A flow chart of the network pharmacology study is shown in **Figure** **1**.

## 3 RESULTS

### 3.1 Candidate targets of dapagliflozin and T2DM complicated with CVD

**FIGURE 2:**
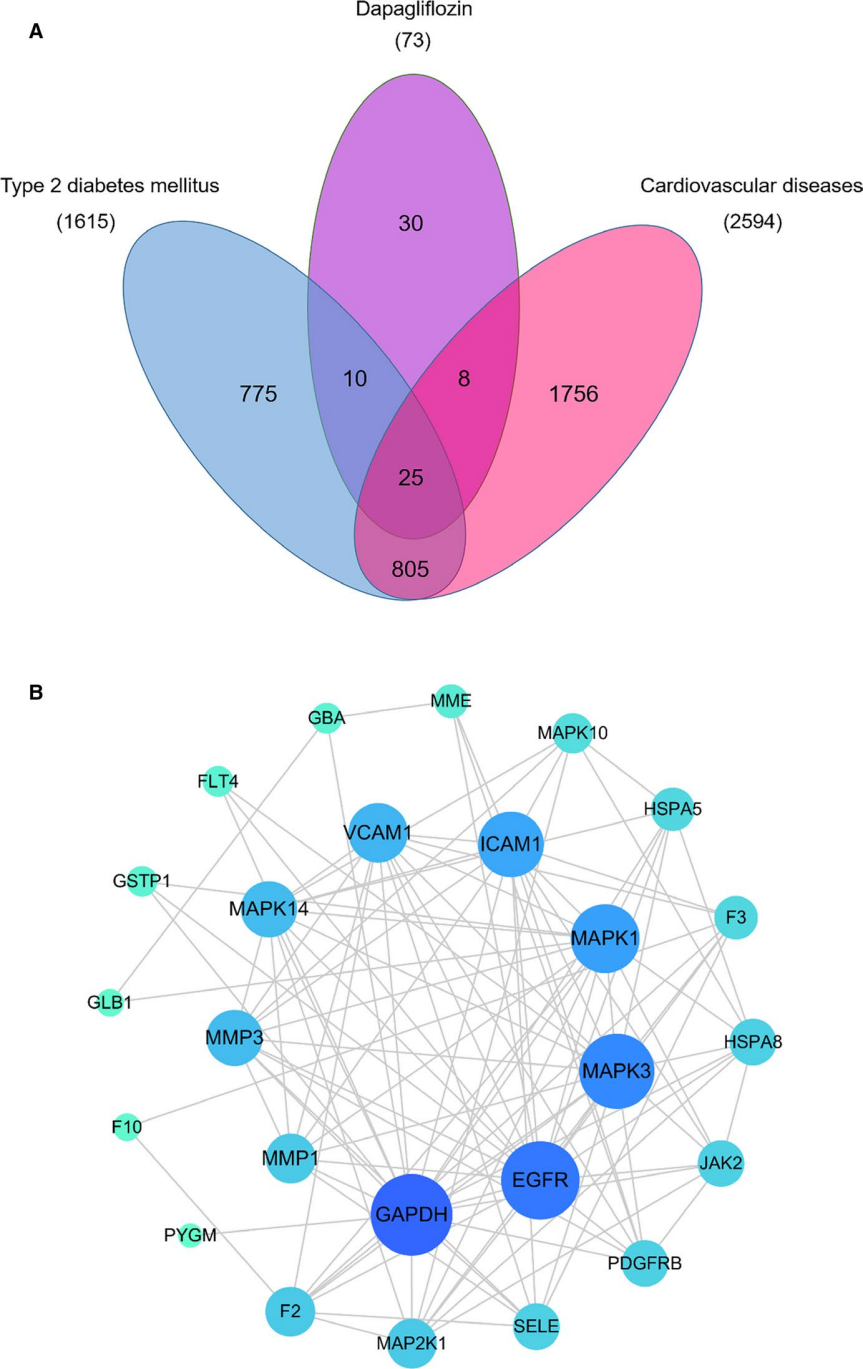
Venn diagram and PPI network. (A) Venn diagram. Blue section stands for T2DM‐related targets, pink section stands for CVD‐related targets, and purple section stands for the potential targets of dapagliflozin. Twenty‐five targets in the middle overlapping section are common targets of dapagliflozin, T2DM and CVD. (B) PPI network. A total of 25 target proteins and 107 interacting edges are in the network. The sizes and colours of the nodes are illustrated from big to small and blue to green in descending order of degree values

After screening the literature and online database, we have identified the bioactive component of dapagliflozin and found 73 candidate genes. The details of these candidate genes are shown in Table S1. For T2DM and its cardiovascular complications, we have retrieved 1505 genes from OMIM database and 4746 genes from Gene Cards database. After removing duplicates data, totally 1615 T2DM‐related genes and 2594 CVD‐related genes were enrolled. To obtain the candidate targets responsible for dapagliflozin in the treatment of T2DM complicated with CVD, we mapped the putative targets of dapagliflozin to the related targets of T2DM complicated with CVD by drawing the Venn diagram, and a total of 25 overlapping targets were obtained (**Figure** **2** **(A)**).

### 3.2 PPI network of T2DM complicated with CVD targets

**FIGURE 3:**
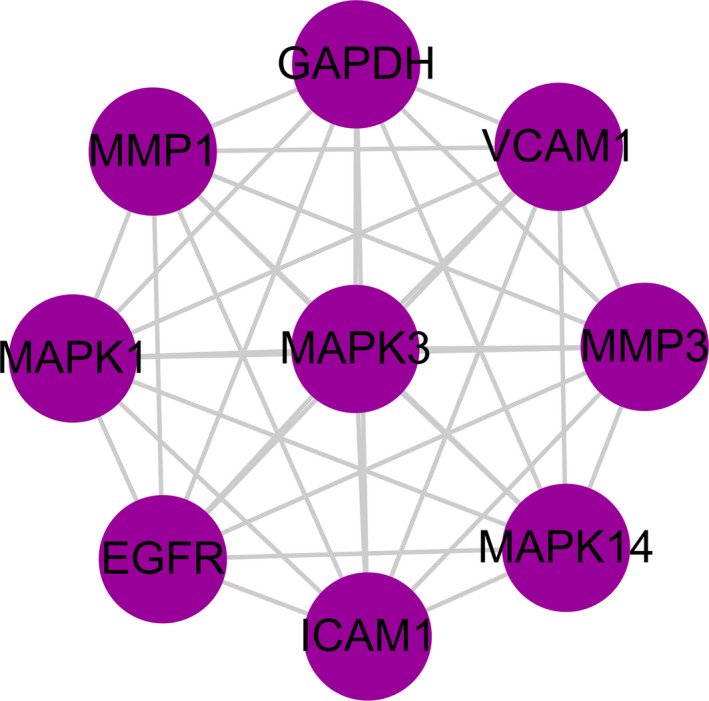
Potential protein functional module with the best score in the PPI network

To estimate the role of the candidate targets in complex disease and found the interactive effects, overlapping targets were submitted to the STRING11.0 platform to establish PPI network, which consisted of 25 targets and 107 interacting edges in total. After analysing the topology parameters of PPI network, the 25 targets were sorted in descending order by degree and arranged in a concentric circle according to the degree (**Figure** **2** **(B)**). In order to more accurately elucidate the mechanism of dapagliflozin in the treatment of T2DM complicated with CVD, we used MCODE to conduct cluster analysis on the PPI network and obtained a potential protein functional module with the best score as shown in **Figure** **3**. It is generally believed that proteins in such potential functional module are more closely related, and they may interact with each other to perform specific biological functions.^23^ Therefore, these proteins might have an important regulatory effect on T2DM complicated with CVD by dapagliflozin.

### 3.3 Possible regulatory signalling pathways of dapagliflozin in treating T2DM complicated with CVD

**FIGURE 4:**
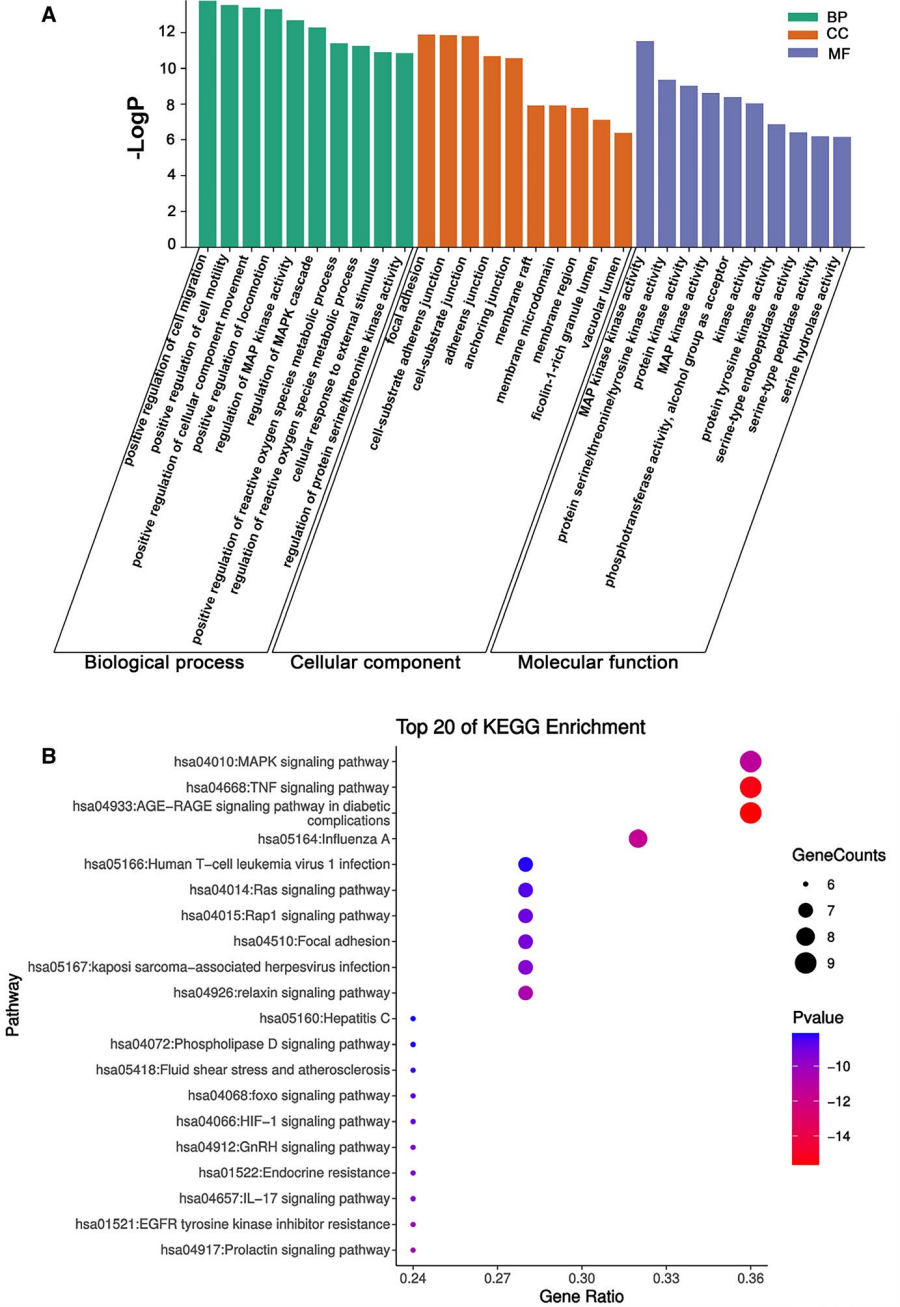
Enrichment analysis of the targets of dapagliflozin in treating T2DM with CVD. (A) GO functional analysis. The top 10 items of each part are shown. (B) KEGG pathway enrichment analysis. The sizes of the bubbles are illustrated from big to small in descending order of the number of the potential targets involved in the pathways

We further conducted GO functional analysis and KEGG pathway enrichment analysis to elucidate the biological effects on gene functions and signalling pathways of the related targets of dapagliflozin in treating T2DM complicated with CVD. As shown in **Figure** **4**, the top 10 GO items and top 20 KEGG pathways were selected based on *p* value.

For molecular functions, it can be seen that the targets were mainly enriched in MAP kinase activity, protein serine/threonine/tyrosine kinase activity, protein kinase activity, MAP kinase activity and phosphotransferase activity (alcohol group as acceptor). In terms of biological processes, the top 10 items were mainly related to positive regulation of cell migration (GO:0 030 335, GO:2 000 147, GO:0 051 272, GO:0 040 017), MAPK cascade (GO:0 043 408, GO:0 043 405), regulation of reactive oxygen species metabolic process (GO:2 000 377, GO:2 000 379), cellular response to external stimulus (GO:0 071 496) and regulation of protein serine/threonine kinase activity (GO:0 071 900). The cellular components were mainly concentrated in focal adhesion, cell‐substrate adheres junction, cell‐substrate junction, adheres junction and anchoring junction. According to the results of the KEGG pathway enrichment analysis, most involved in MAPK signalling pathway, TNF signalling pathway, AGE‐RAGE signalling pathway in diabetic complications, influenza A, human T‐cell leukaemia virus 1 infection, Ras signalling pathway, Rap1 signalling pathway, focal adhesion, kaposi sarcoma‐associated herpesvirus infection and relaxin signalling pathway. Interestingly, the common targets of dapagliflozin, T2DM and CVD mainly enriched in MAPK signalling pathway and two of the top 10 biological process items were related to MAPK cascade. In addition, most of the molecular function items focused on regulating kinase activity, implying that the MAPK signalling pathway might be the key pathway.

### 3.4 Component‐target‐pathway network construction

**FIGURE 5:**
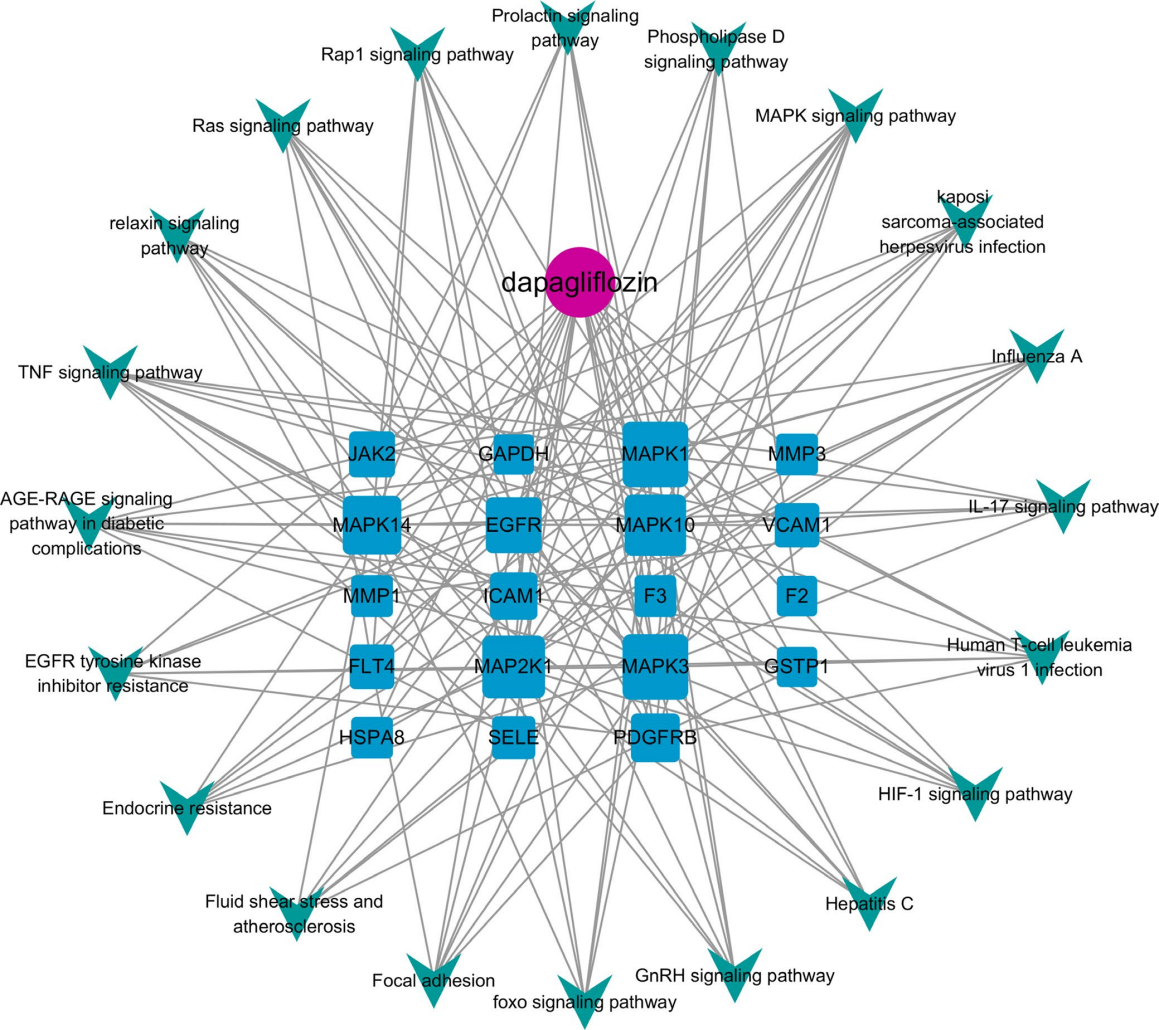
Component‐target‐pathway network. A total of 40 nodes and 156 edges are in the network. The purple circle represents the bioactive component of dapagliflozin, 19 blue squares represent targets, and 20 green V‐shapes represent pathways. The sizes of the blue square node are illustrated from big to small in descending order of degree values. One hundred and fifty‐six edges represent the interaction relationship between component, targets and pathways

Component‐target‐pathway network was constructed with Cytoscape3.7.2 based on the results of KEGG pathway enrichment analysis (**Figure** **5**). Degree reflected the importance of nodes by representing the number of connections between nodes and other nodes. The average degree of the targets included in the network was 8.21, and there were 6 targets with degree higher than 8.21, namely MAPK1 (degree =20), MAPK3 (degree =20), MAP2K1 (degree =18), MAPK10 (degree =17), MAPK14 (degree =15) and EGFR (degree =13). As MAPK1, MAPK3, MAPK14 and EGFR were also in the protein functional module with the best score, we speculated that the 4 targets might be the key targets for dapagliflozin in the treatment of T2DM complicated with CVD.

### 3.5 Molecular docking results analysis

**FIGURE 6:**
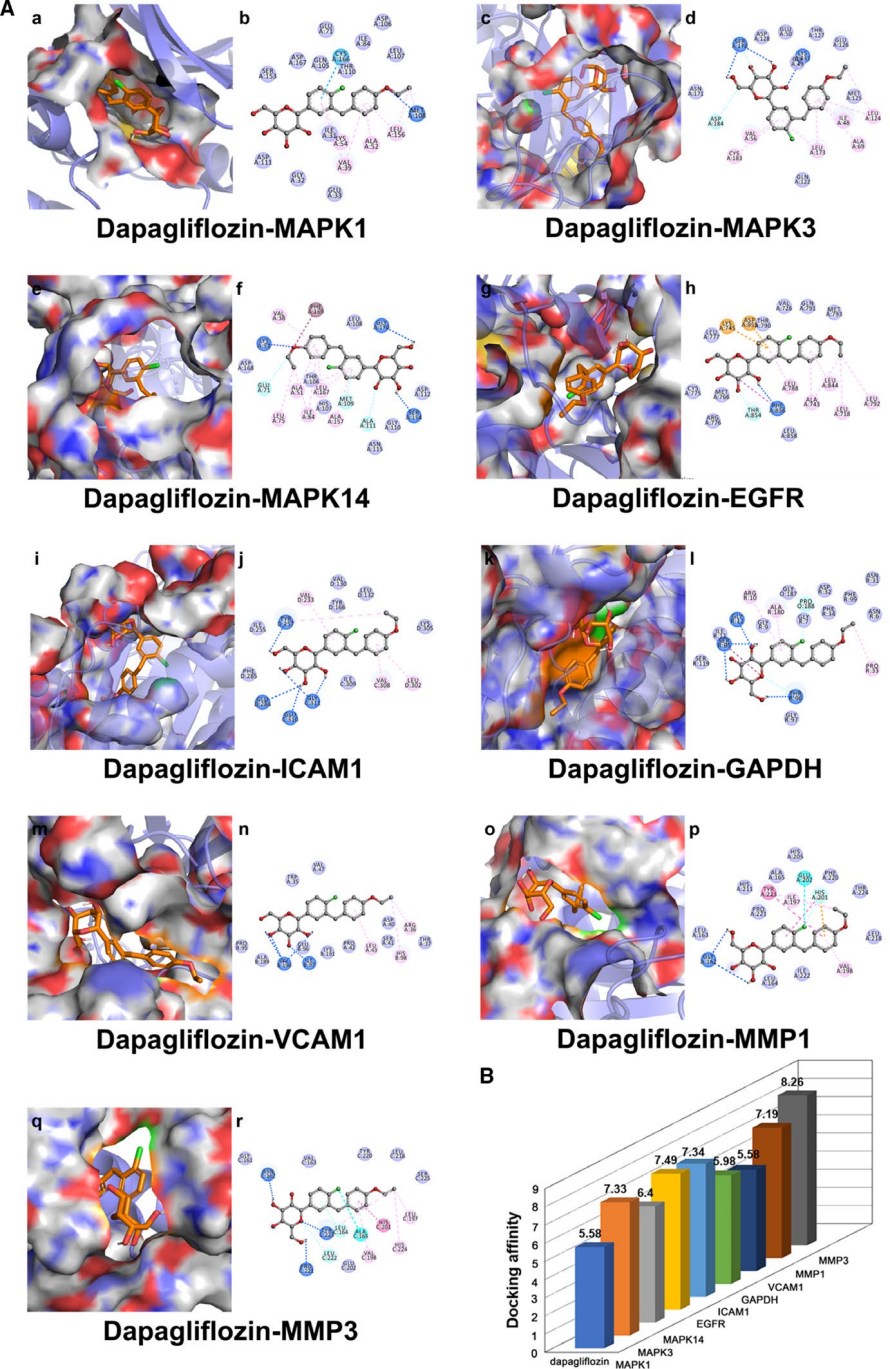
Molecular docking diagram. (A) Nine conformations of molecular docking simulation. Diagrams (3D) represent that molecular model of the compound is in the binding pocket of the protein. The compound is shown as stick model with orange coloured. The amino acid residues surrounding are represented by surface style. Diagrams (2D) show the interactions between compound and surrounding residues. (B) 3D column diagram shows the affinity of 9 conformations. X‐axis: bioactive component, y‐axis: target names, z‐axis: docking affinity (absolute value of the binding energy)

In the present study, the interaction activity between the core genes and dapagliflozin was verified by molecular docking. It is generally believed that the lower the energy when the conformation of ligand binding to the receptor is stable, the greater the possibility of interaction. Totally, nine pairs were delivered into the docking simulation, and all binding complexes showed good binding affinity with an average of −6.8 kcal/mol. Nine conformations with the lowest binding energy were visualized, including dapagliflozin‐MAPK1 docking (−5.58 kcal/mol), dapagliflozin‐MAPK3 docking (−7.33 kcal/mol), dapagliflozin‐MAPK14 docking (−6.4 kcal/mol), dapagliflozin‐EGFR docking (−7.49 kcal/mol), dapagliflozin‐ICAM1 docking (−7.34 kcal/mol), dapagliflozin‐GAPDH docking (−5.98 kcal/mol), dapagliflozin‐VCAM1 docking (−5.58 kcal/mol), dapagliflozin‐MMP1 docking (−7.19 kcal/mol) and dapagliflozin‐MMP3 docking (−8.26 kcal/mol) (**Table** **S2**, **Figure** **6**). Specifically, taking the dapagliflozin‐MAPK1 docking for example, small molecule ligand dapagliflozin potentially fitted into the interface pocket formed by interaction amino acid residues in protein (**Figure** **6A** **(a)**). As shown in Figure 6A(b), hydrogen bond was formed between dapagliflozin and MET108 near the active site of MAPK1. The other essential residues (SER153, ASP167, ASP111, GLN105, THR110, GLU33, GLU71, ILE84, ILE31, LEU107, ASP106, GLY32, LYS54, VAL39, ALA52, LEU156 and CYS166) interacted with dapagliflozin through van der Waals forces, pi‐alkyl interaction and pi‐donor hydrogen bond. These hydrogen bonds and interaction forms contributed to the stable binding of small molecules to the active sites of their respective proteins.

## 4 DISCUSSION

The combination of network pharmacology and molecular docking technology has been widely used to elucidate the relationship between the bioactive components of a drug and their potential mechanism of action. In the present study, we used network pharmacology method and molecular docking technology to explore the multi‐target and multi‐pathway therapeutic mechanism of dapagliflozin in the treatment of T2DM complicated with CVD. After integrating and collating information from several available databases, dapagliflozin acted on a total of 25 targets associated with T2DM and CVD. Further, to better explore the mechanism of dapagliflozin in treating T2DM complicated with CVD, a potential protein functional module with the best score was identified from the PPI network of the 25 targets. These targets in the protein functional module were more closely linked, forming complexes that worked together biologically. The biological process of the protein functional module was mainly involved in chemotaxis and migration of macrophages, which reflected the pathogenesis of T2DM and CVD to some extent and was consistent with previous studies.

The pathological process of T2DM is often accompanied by local tissue inflammation, which is characterized by macrophage infiltration. Recent studies have proved that macrophages play multiple roles in the occurrence and development of CVD and are involved in the pathological changes and tissue remodelling of CVD such as arteriosclerosis and myocardial infarction.^24^, ^25^ Notably, four targets in the protein functional module were included in the key targets which were screened out in the component‐target‐pathway network, reconfirming the importance of the protein functional module in the therapeutic mechanism of dapagliflozin on T2DM complicated with CVD.

**FIGURE 7:**
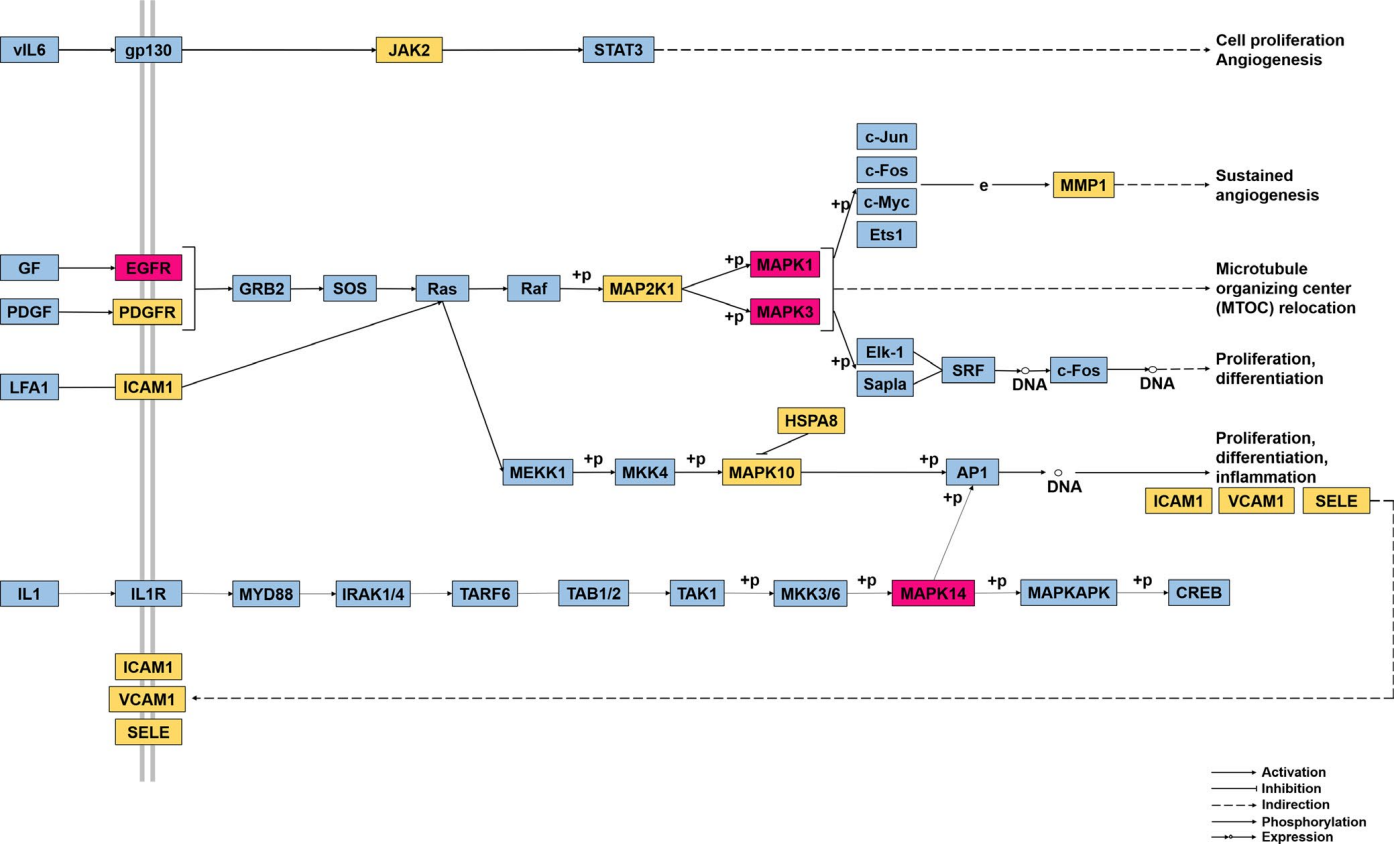
MAPK signalling pathway influenced by dapagliflozin. The red nodes represent the key targets, the yellow nodes represent common targets of dapagliflozin, T2DM and CVD targets, and the blue nodes represent the other targets of this pathway. Dapagliflozin mainly affects the classical MAPK signalling pathway and p38/MAPK signalling pathway. As for the classical MAPK signalling pathway, dapagliflozin acts on EGFR and PDGFR on the cell membrane, affecting the activation of GRB2, SOS, Ras, Raf, MAP2K1 and phosphorylation of MAPK1 and MAPK3 and finally causing changes in angiogenesis, cell apoptosis and cell proliferation. In the p38/MAPK signalling pathway, dapagliflozin acts on MAPK14, and when MAPK14 enters the nucleus, it affects the transcription and then regulates cell survival, proliferation, differentiation and other processes

According to KEGG pathway enrichment analysis and literature review, we hypothesized that dapagliflozin might play a therapeutic role in T2DM complicated with CVD mainly through the regulation of the MAPK signalling pathway **(** **Figure** **7** **)**. Based on the analysis of the component‐target‐pathway network, we screened MAPK1, MAPK3, MAPK14 and EGFR as key targets, indicating that the role of dapagliflozin‐mediated therapy in the treatment of T2DM complicated with CVD might be mainly associated with above targets. For instance, MAPK1, as a key kinase of cell stress transmission, is involved in the regulation of a variety of cellular functions and is related to hyperglycaemia, oxidative stress and growth factors in diabetic. MAPK signalling cascade also plays a vital role in the pathogenesis of CVD, such as cardiac remodelling after myocardial infarction, the formation of atherosclerosis and the formation of neovascularization, and its imbalance will lead to the occurrence of corresponding CVD.^26^ Functional SGLT2 is expressed in intact vascular tissue and may be a major target of SGLT2 inhibitors in blood vessels. It is generally recognized that endothelial dysfunction caused by hyperglycaemia is the main factor of cardiovascular complications in T2DM.^27^ Hyperglycaemia triggers increased intracellular glucose oxidation mediated by SGLT2, which leads to endothelial signalling via EGFR kinase triggered by reactive oxygen species (ROS), and ultimately to endothelial dysfunction.^28^ SGLT2 inhibitors may protect endothelial cells from increased glucose uptake and ROS production, inhibit EGFR kinase activity and thus prevent high glucose‐induced endothelial dysfunction and diabetic cardiovascular complications.^28^, ^29^ Coincidentally, all the above key targets were enriched in MAPK signalling pathway, which was consistent with our hypothesis. Subsequently, through molecular docking, the results that dapagliflozin docked well with core genes in the protein functional module further supported the correctness of our conjecture. Therefore, it is vital to investigate the role of MAPK signalling pathway in dapagliflozin in the treatment of T2DM complicated with CVD.

SGLT2 is mainly located in the epithelial cells of the S1 and S2 segments of the renal proximal tubule, responsible for the reabsorption of about 90% glucose in urine.^30^ Besides this, there are a large number of mitochondria in the proximal renal tubules, which can meet the energy requirements of SGLT2 reabsorption task and maintain its high metabolic function.^31^, ^32^ In patients with T2DM, the expression and activity of SGLT2 are both increased, leading to an increase in the amount of glucose reabsorbed.^33^ Although the number of studies on MAPK signalling pathway in T2DM is limited, the available evidence suggests that MAPK act in a signalling cascade that regulates SGLT2 activity. Phosphorylation of p38 MAPK is considered as an important signal transduction step to regulate the activity of SGLT2, and cyclic adenosine monophosphate (cAMP) induced cAMP‐activated guanine nucleotide exchange factor and protein kinase A activation increase SGLT2 expression and trafficking through p38 MAPK phosphorylation.^34^, ^35^ In addition, ATP induces phosphorylation of p38 MAPK and stimulates uptake of [14C]‐α‐methyl‐D‐glucopyranoside in renal proximal tubule cells via cAMP and p38 MAPK.^36^ Consequently, it can be concluded from the existing researches that MAPK signalling pathway plays a key role in the expression and transport of SGLT2, and inhibiting this pathway may be an effective way for dapagliflozin against T2DM.

Up till now, there has been considerable clinical evidence that dapagliflozin as a SGLT2 inhibitor significantly improves cardiovascular outcome in patients with T2DM. Interestingly, however, no expression of SGLT2 was detected in the human heart,^37^, ^38^ which intrigued us even more. MAPK signalling pathway is widely associated with cardiac pathology, and changes in MAPK expression and activity affect the occurrence and development of CVD. In the hearts of diabetic patients, the cardiac activity of ERK1/2, JNK and p38 MAPK is enhanced and participates in the development of myocardial remodelling.^39^ Research proved that dapagliflozin could significantly reduce the phosphorylation levels of JNK and p38 MAPK in mouse cardiomyocytes, improved cardiac hypertrophy and myocardial interstitial fibrosis and delayed the pathological progression of ventricular systolic dysfunction.^40^ Similarly, in myocardial ischaemia models, SGLT2 inhibitor not only prevented left ventricular remodelling and fibrosis area expansion after myocardial ischaemia, but also protected the myocardial damage caused by ischaemia reperfusion.^41^, ^42^ One of the pathological basis of the coronary heart disease is coronary atherosclerosis. In vitro experiment has indicated that SGLT2 inhibitor can inhibit the activation of p38 MAPK and has a protective effect on vascular endothelial cells, thereby reducing the development of atherosclerotic lesions.^43^ Moreover, under normal conditions, mitochondria directly or indirectly related to the MAPK signalling pathway, producing about 80% of the ATP needed for cardiomyocytes, and the heart is also flexible enough to use different substrates to produce energy.^44^, ^45^ However, mitochondrial function and dynamics are impaired in type 2 diabetics, leading to myocardial systolic dysfunction.^46^, ^47^ On the other hand, the metabolic flexibility of the heart is impaired in type 2 diabetic patients, and heart failure may occur when the energy of the heart is not sufficient to maintain its function.^45^, ^48^ Uthman et al recently proved that SGLT2 inhibitors played a direct role in combating heart failure by inhibiting myocardial mitochondrial Na^+^/H^+^ exchanger flux and subsequently reducing intracellular free Na^+^, which was consistent with Durak and coworkers’ findings.^49^, ^50^ In diabetic mice with cardiac dysfunction were treated with SGLT2 inhibitor, improvements were observed in the myocardial mitochondrial ultrastructure and sarcomere organization.^51^ Altogether, SGLT2 inhibitor optimizes mitochondrial function and energy metabolism in cardiomyocytes, which is beneficial to the improvement of cardiac function to some extent. Therefore, we suspect that the MAPK signalling pathway may be an indispensable pathway for SGLT2 inhibitor to regulate energy metabolism of cardiomyocytes in patients with T2DM.

**FIGURE 8:**
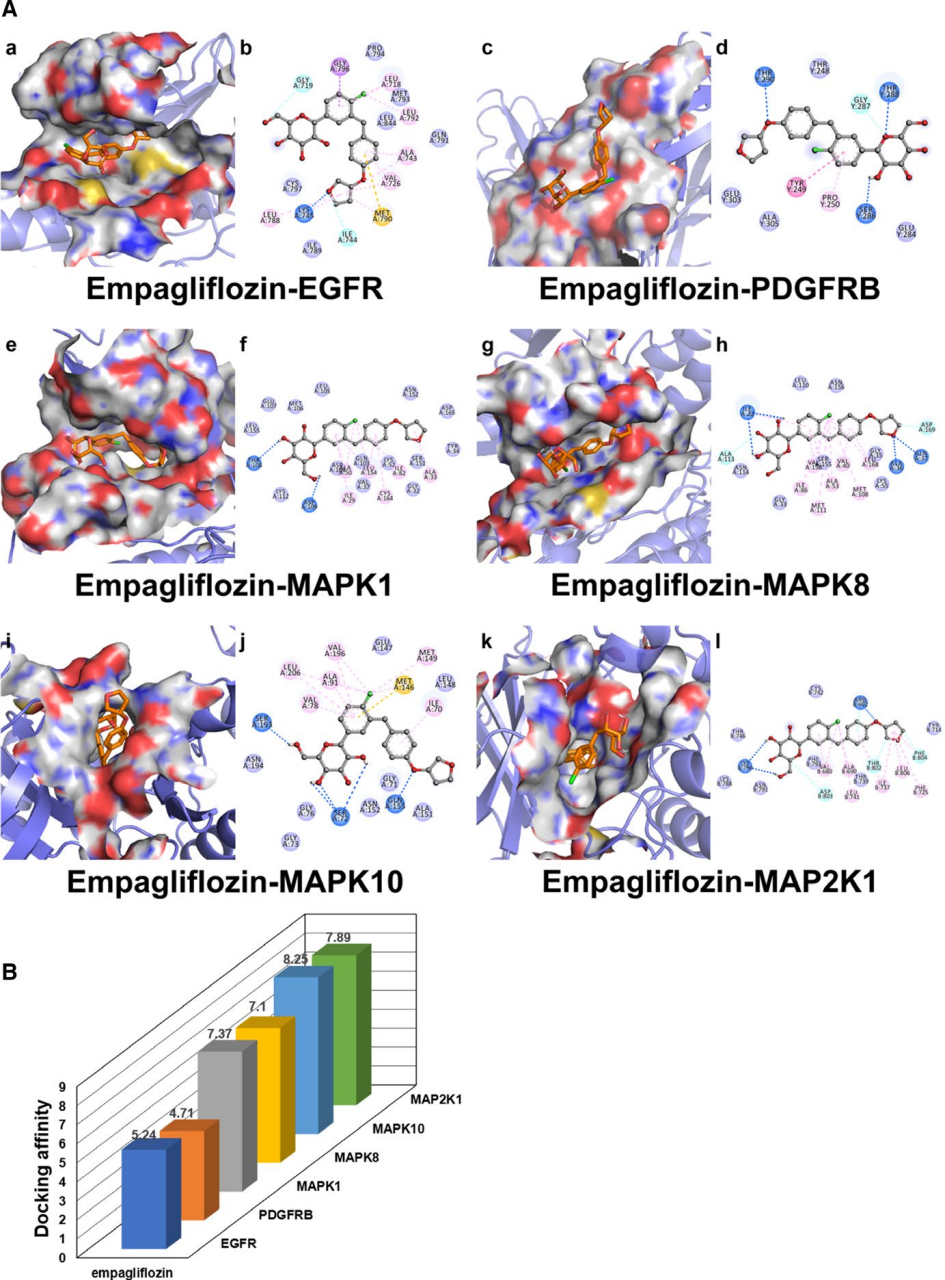
Molecular docking diagram of empagliflozin docking with common targets enriched in the MAPK signalling pathway. (A) Six conformations of molecular docking simulation. (B) 3D column diagram shows the affinity of 6 conformations

**FIGURE 9:**
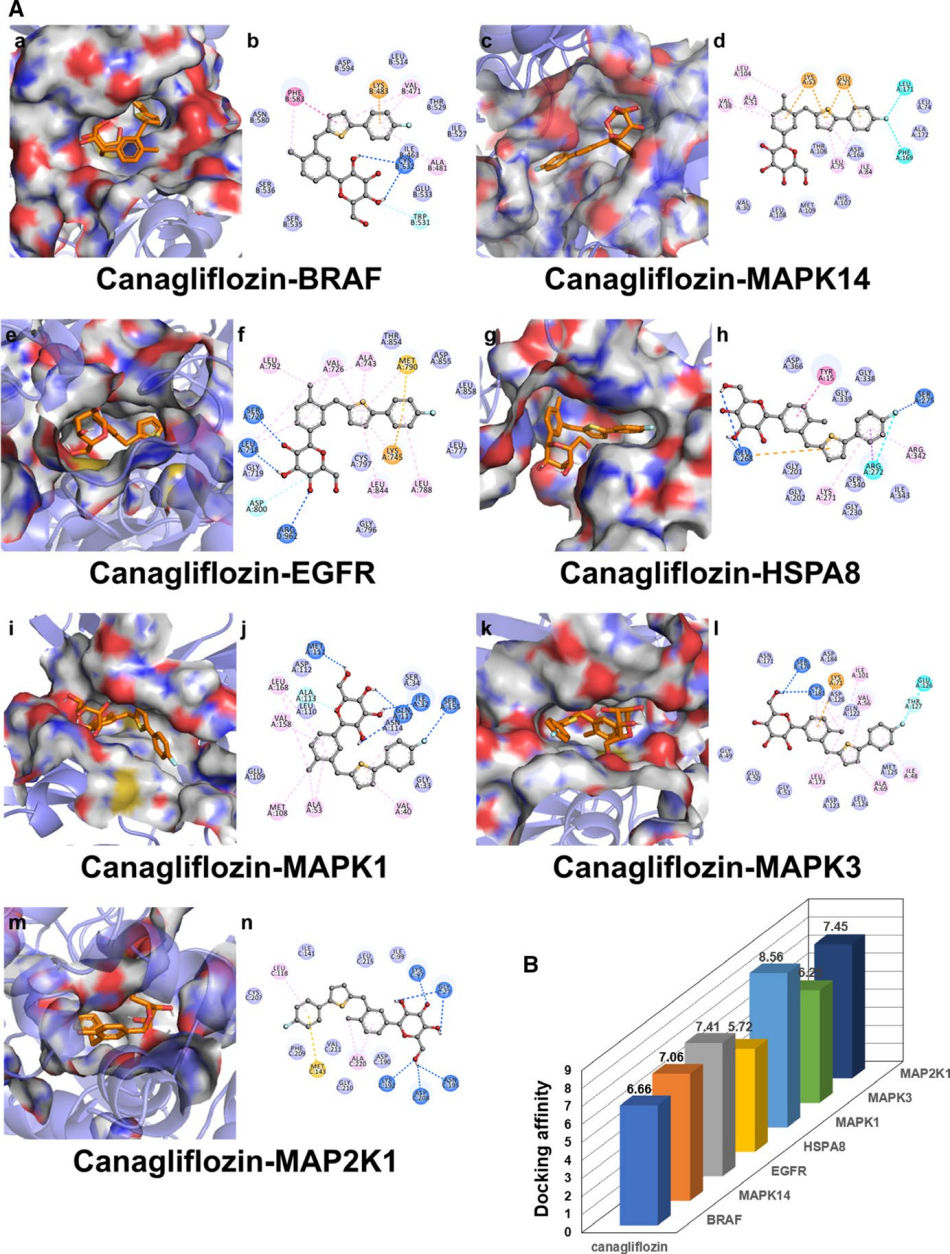
Molecular docking diagram of canagliflozin docking with common targets enriched in the MAPK signalling pathway. (A) Seven conformations of molecular docking simulation. (B) 3D column diagram shows the affinity of 7 conformations

In addition to dapagliflozin, other SGLT2 inhibitors have also been demonstrated to have cardiovascular protective effects.^52^, ^53^, ^54^, ^55^, ^56^ A recent meta‐analysis conducted by Zelniker et al suggested that SGLT2 inhibitors, including dapagliflozin, empagliflozin and canagliflozin, significantly reduced the risk of cardiovascular death or hospitalization for heart failure and in particular apparently reduced major cardiac adverse events in patients with atherosclerotic CVD. Although there were some differences in specific effects between different SGLT2 inhibitors, they were generally well tolerated and safe.^52^ This study provides highly convincing evidence of the cardiovascular protective effects of SGLT2 inhibitors. With this in mind, we used the same network pharmacological approach to validate two common SGLT2 inhibitors, namely, empagliflozin and canagliflozin, to determine whether their benefit in T2DM complicated with CVD is also related to the MAPK signalling pathway. Remarkably, we found that MAPK signalling pathway was one of the important pathways in which the common targets of these inhibitors and T2DM complicated with CVD were enriched, and the biological processes were mainly involved in the regulation of MAPK kinase activity. Molecular docking results also showed that the bioactive components of the two inhibitors had good binding capacity with the common targets enriched in the MAPK signalling pathway, respectively (**Table** **S3**, **Table** **S4**). **Figure** **8** and **Figure** **9** were visualizations of the conformations with the lowest binding energy. This suggests that SGLT2 inhibitors have some similarities in the therapeutic mechanism of T2DM complicated with CVD, and it is necessary to conduct more in depth studies on their mechanism. Targeting MAPK signalling pathway may lead to novel therapeutic strategies for patients with T2DM complicated with CVD.

In conclusion, our research systematically elucidated the underlying molecular mechanisms by which dapagliflozin simultaneously interfered with T2DM and CVD based on the network pharmacology and molecular docking. We predicted 4 key targets from complex networks and concluded that the MAPK signalling pathway is the main pathway for dapagliflozin in the treatment of T2DM complicated with CVD. We also preliminarily predicted that empagliflozin and canagliflozin interacted well with targets on the MAPK pathway. The above results provided scientific evidence for clinical application of dapagliflozin in the treatment of T2DM complicated and CVD, and also provided a new idea for the future research on the mechanism of action of SGLT2 inhibitors.

## CONFLICT OF INTEREST

The authors declare that they have no known competing financial interests or personal relationships that could have appeared to influence the work reported in this paper.

## AUTHOR CONTRIBUTIONS

**Zhaodi Yue:** Conceptualization (equal); Validation (equal); Writing‐original draft (lead); Writing‐review & editing (equal). **Li Li:** Conceptualization (equal). **Hui Fu:** Data curation (equal); Investigation (equal). **Yanyan Yin:** Data curation (equal); Investigation (equal). **Bingyu Du:** Data curation (equal); Investigation (equal). **Fangqi Wang:** Data curation (equal); Investigation (equal). **Yi Ding:** Writing‐review & editing (equal). **Yibo Liu:** Software (equal); Visualization (equal). **Renjie Zhao:** Software (equal); Visualization (equal). **Zhongwen Zhang:** Conceptualization (lead); Funding acquisition (lead); Supervision (equal); Writing‐review & editing (equal). **Shaohong Yu:** Conceptualization (lead); Formal analysis (lead); Supervision (equal).

## Supporting information

Table S1

Table S2

Table S3

Table S4

## Data Availability

The data that support the findings of this study are available in the supplementary material of this article.
