# Supplementary material for: Effect of dapagliflozin on diabetic patients with cardiovascular disease via MAPK signalling pathway
Source: J Cell Mol Med. 2021 Jul 14;25(15):7500–12. doi: 10.1111/jcmm.16786 (PMC8335696; doi:10.1111/jcmm.16786)
Supplement: Supplementary file 1 — Table S1 [file JCMM-25-7500-s002.docx]

**Table S1** **The basic information of 73 predicted and validated targets of dapagliflozin.**

| Target name | Gene name | Uniprot ID | Probability |
| --- | --- | --- | --- |
| Sodium/myo-inositol cotransporter 2  Solute carrier family 5 member 4  Sodium/glucose cotransporter 2  Sodium/glucose cotransporter 1  Adenosine kinase  Platelet-derived growth factor receptor beta  Tyrosine-protein kinase LCK  Mitogen-activated protein kinase 14  Mitogen-activated protein kinase 10  Mitogen-activated protein kinase 11  Ephrin type-A receptor 5  Cyclin-G-associated kinase  Adenosine receptor A2a  Adenosine receptor A3  Dual specificity mitogen-activated protein kinase kinase 1  Equilibrative nucleoside transporter 1  Non-lysosomal glucosylceramidase  Lysosomal acid glucosylceramidase  Sodium/nucleoside cotransporter 2  Heat shock cognate 71 kDa protein  Endoplasmic reticulum chaperone BiP  Hexokinase-2  Hexokinase-1  Phosphodiesterase 5A  Tissue factor  Glycogen phosphorylase, liver form  Uridine phosphorylase 1  Adenosine A2b receptor  Procathepsin L  Glutathione S-transferase P  Glutathione S-transferase Mu 2  Solute carrier family 2, facilitated glucose transporter member 1  Glycogen phosphorylase, muscle form  Gamma-aminobutyric acid receptor subunit alpha-5  Epidermal growth factor receptor  Alkaline phosphatase, tissue-nonspecific isozyme  Glyceraldehyde-3-phosphate dehydrogenase  Stromelysin-1  Interstitial collagenase  Serine/threonine-protein kinase Chk1  Protein O-GlcNAcase  P2Y purinoceptor 12  Glutamate receptor ionotropic, NMDA 1  Interleukin-1 receptor-associated kinase 4  Mitogen-activated protein kinase kinase kinase 14  Tyrosine-protein kinase JAK2  Carbonic anhydrase 14  Tissue alpha-L-fucosidase  Intercellular adhesion molecule 1  Vascular cell adhesion protein 1  E-selectin  Vascular endothelial growth factor receptor 3  Neprilysin  Glutamate receptor ionotropic, kainate 1  Beta-galactosidase  Mitogen-activated protein kinase 1  Endothelin-converting enzyme 1  Prothrombin  Coagulation factor X  Cyclin-dependent kinase 1  G2/mitotic-specific cyclin-B1  Cyclin-dependent kinase 2  G1/S-specific cyclin-E1  Cyclin-dependent kinase 7  Cyclin-H  Cyclin-dependent kinase 9  Cyclin-T1  Dual specificity tyrosine-phosphorylation- regulated kinase 1A  Casein kinase I alpha  Mitogen-activated protein kinase 3  Mitogen-activated protein kinase 15  Calcium/calmodulin-dependent protein kinase kinase 2  Insulin receptor-related protein | SLC5A11  SLC5A4  SLC5A2  SLC5A1  ADK  PDGFRB  LCK  MAPK14  MAPK10  MAPK11  EPHA5  GAK  ADORA2A  ADORA3  MAP2K1  SLC29A1  GBA2  GBA  SLC28A2  HSPA8  HSPA5  HK2  HK1  PDE5A  F3  PYGL  UPP1  ADORA2B  CTSL  GSTP1  GSTM2  SLC2A1  PYGM  GABRA5  EGFR  ALPL  GAPDH  MMP3  MMP1  CHEK1  OGA  P2RY12  GRIN1  IRAK4  MAP3K14  JAK2  CA14  FUCA1  ICAM1  VCAM1  SELE  FLT4  MME  GRIK1  GLB1  MAPK1  ECE1  F2  F10  CDK1  CCNB1  CDK2  CCNE1  CDK7  CCNH  CDK9  CCNT1  DYRK1A  CSNK1A1  MAPK3  MAPK15  CAMKK2  INSRR | Q8WWX8  Q9NY91  P31639  P13866  P55263  P09619  P06239  Q16539  P53779  Q15759  P54756  O14976  P29274  P0DMS8  Q02750  Q99808  Q9HCG7  P04062  O43868  P11142  P11021  P52789  P19367  O76074  P13726  P06737  Q16831  P29275  P07711  P09211  P28161  P11166  P11217  P31644  P00533  P05186  P04406  P08254  P03956  O14757  O60502  Q9H244  Q05586  Q9NWZ3  Q99558  O60674  Q9ULX7  P04066  P05362  P19320  P16581  P35916  P08473  P39086  P16278  P28482  P42892  P00734  P00742  P06493  P14635  P24941  P24864  P50613  P51946  P50750  O60563  Q13627  P48729  P27361  Q8TD08  Q96RR4  P14616 | 1  1  1  1  0.1157  0.1157  0.1157  0.1157  0.1157  0.1157  0.1157  0.1157  0.1157  0.1157  0.1157  0.1157  0.1157  0.1157  0.1157  0.1157  0.1157  0.1157  0.1157  0.1157  0.1157  0.1157  0.1157  0.1157  0.1157  0.1157  0.1157  0.1157  0.1157  0.1157  0.1157  0.1157  0.1157  0.1157  0.1157  0.1157  0.1157  0.1157  0.1157  0.1157  0.1157  0.1157  0.1157  0.1157  0.1157  0.1157  0.1157  0.1157  0.1157  0.1157  0.1157  0.1157  0.1157  0.1157  0.1157  0.1157  0.1157  0.1157  0.1157  0.1157  0.1157  0.1157  0.1157  0.1157  0.1157  0.1157  0.1157  0.1157  0.1157 |
