# Supplementary material for: Effect of dapagliflozin on diabetic patients with cardiovascular disease via MAPK signalling pathway
Source: J Cell Mol Med. 2021 Jul 14;25(15):7500–12. doi: 10.1111/jcmm.16786 (PMC8335696; doi:10.1111/jcmm.16786)
Supplement: Supplementary file 2 — Table S2 [file JCMM-25-7500-s003.docx]

**Table S2** **The binding energy of the dapagliflozin with MAPK1, MAPK3, MAPK14, EGFR, ICAM1, GAPDH, VCAM1, MMP1 and MMP3.**

| Bioactive component | Target name | PDB ID | Binding energy (kcal/mol) |
| --- | --- | --- | --- |
| Dapagliflozin | MAPK1 | 5K4I | -5.58 |
|  | MAPK3 | 2ZOQ | -7.33 |
|  | MAPK14 | 3FLY | -6.40 |
|  | EGFR  ICAM1  GAPDH  VCAM1  MMP1  MMP3 | 3POZ  1RD4  4O59  1VSC  1CXV  1G4K | -7.49  -7.34  -5.98  -5.58  -7.19  -8.26 |
