# Supplementary material for: Effect of dapagliflozin on diabetic patients with cardiovascular disease via MAPK signalling pathway
Source: J Cell Mol Med. 2021 Jul 14;25(15):7500–12. doi: 10.1111/jcmm.16786 (PMC8335696; doi:10.1111/jcmm.16786)
Supplement: Supplementary file 3 — Table S3 [file JCMM-25-7500-s001.docx]

**Table S3** **The binding energy of the empagliflozin with EGFR, PDGFRB, MAPK1, MAPK8, MAPK10 and MAP2K1.**

| Compound | Target name | PDB ID | Binding energy (kcal/mol) |
| --- | --- | --- | --- |
| Empagliflozin | EGFR | 3W2P | -5.24 |
|  | PDGFRB | 3MJG | -4.71 |
|  | MAPK1 | 4ZZN | -7.37 |
|  | MAPK8  MAPK10  MAP2K1 | 2NO3  2B1P  5KKR | -7.10  -8.25  -7.89 |
