# Supplementary material for: Effect of dapagliflozin on diabetic patients with cardiovascular disease via MAPK signalling pathway
Source: J Cell Mol Med. 2021 Jul 14;25(15):7500–12. doi: 10.1111/jcmm.16786 (PMC8335696; doi:10.1111/jcmm.16786)
Supplement: Supplementary file 4 — Table S4 [file JCMM-25-7500-s004.docx]

**Table S4** **The binding energy of the canagliflozin with BRAF, MAPK14, EGFR, HSPA8, MAPK1, MAPK3 and MAP2K1.**

| Compound | Target name | PDB ID | Binding energy (kcal/mol) |
| --- | --- | --- | --- |
| Canagliflozin | BRAF | 4CQE | -6.66 |
|  | MAPK14 | 1A9U | -7.06 |
|  | EGFR  HSPA8  MAPK1  MAPK3  MAP2K1 | 6V5N  3FZL  3PZE  6GES  7JUZ | -7.41  -5.72  -8.56  -6.25  -7.45 |
